# The influence of psychological stress in early life on sexual maturation and sexual behavior in male and female rats

**DOI:** 10.1002/rmb2.12313

**Published:** 2019-12-26

**Authors:** Kiyohito Yano, Toshiya Matsuzaki, Takeshi Iwasa, Yiliyasi Mayila, Rie Yanagihara, Altankhuu Tungalagsuvd, Munkhsaihan Munkhzaya, Takako Tokui, Shuhei Kamada, Aki Hayashi, Rie Masaki, Hidenori Aoki, Kou Tamura, Minoru Irahara

**Affiliations:** ^1^ Department of Obstetrics and Gynecology Graduate School of Biomedical Sciences Tokushima University Tokushima Japan; ^2^ Division of Obstetrics and Gynecology Yoshinogawa Medical Center Tokushima Japan; ^3^ Division of Obstetrics and Gynecology National Center for Maternal and Child Health Ulaanbaatar Mongolia; ^4^ Department of Gynecology The First Maternity Hospital of Mongolia Ulaanbaatar Mongolia

**Keywords:** early‐life stress, maternal separation, psychological stress, sexual behavior, sexual maturation

## Abstract

**Purpose:**

We studied the influence of psychological stress during the early neonatal period on sexual maturation and sexual behavior in rats.

**Methods:**

Neonatal male and female rats were divided into control (C) and maternal separation (MS) groups (n = 20‐24 per group). The pups in the MS groups were placed in isolation cages for 240 minutes/d from postnatal days 2‐11. Vaginal opening (VO) in females and preputial separation (PS) in males (indicators of sexual maturation) were monitored, as was the estrous cycle in females. Thereafter, sexual behavior was monitored twice at 13 and 15 weeks of age.

**Results:**

As for sexual maturation, the onset of PS occurred significantly earlier in the MS group than in the C group, whereas the onset of VO did not differ between the groups. The length of the estrous cycle did not differ between the groups. The frequencies of sexual behaviors did not differ between the groups in either sex.

**Conclusions:**

In conclusion, early‐life psychological stress induced by MS advanced sexual maturation in male rats, whereas it did not affect sexual maturation in female rats. On the other hand, early‐life psychological stress might not affect sexual behavior in adulthood in either sex.

## 1 INTRODUCTION

It has been reported that several kinds of stress that occur during the perinatal period have a long‐lasting influence on physiological and psychological functions. For example, in humans the prevalence rates of hyperlipidemia, coronary heart disease, renal damage, diabetes, and schizophrenia were higher among the children of undernourished mothers than among the children of normally nourished mothers.^1^, ^2^, ^3^ In addition, the cognitive and reading abilities of the children of undernourished mothers were also lower than those of the children of normally nourished mothers.^4^, ^5^ Furthermore, in rats we have shown that sexual maturation was disturbed in both the male and female offspring of undernourished mothers.^6^, ^7^, ^8^ These findings indicate that prenatal nutritional stress has adverse effects on physiological, psychological, and reproductive functions, and these changes might cause certain diseases in later life. It has also been reported that immune stress in childhood increases the risk of cancer, lung disease, and arthritis/rheumatism and alters cardiovascular functions in later life in humans.^9^ Furthermore, we have shown that neonatal immune stress increased appetite and body weight gain in male rats and disturbed the estrous cycle in female rats in adulthood.^6^, ^10^ In addition, Walker et al and we have shown that neonatal immune stress impaired sexual maturation and sexual behavior in male and female rats.^11^, ^12^ These findings suggest that perinatal immune stress, as well as nutritional stress, affects metabolic and reproductive functions in later life.

Neonatal psychological stress might be an independent risk factor for the development of childhood obesity in humans.^13^ This finding has also been reproduced in animal models, for example, neonatal maternal separation (MS) and early weaning‐induced hyperinsulinemia and glucose intolerance in adulthood in female mice.^14^ On the contrary, to the best of our knowledge, the effects of neonatal psychological stress on reproductive functions in later life have not been investigated in detail. Only one study has examined the effects of neonatal psychological stress on sexual function, and it only focused on female sexual maturation/estrous cyclicity and male sexual behavior.^15^ Therefore, in the present study we examined the influence of early‐life psychological stress induced by MS on sexual maturation and sexual behavior in later life in both sexes.

## 2 MATERIALS AND METHODS

### 2.1 Animals

Six pregnant Sprague Dawley rats were purchased (Charles River Japan, Inc) and housed individually under controlled lighting (14‐hour light, 10‐hour darkness) and temperature (24°C) conditions. All animal experiments were conducted in accordance with the ethical standards of Tokushima University. The day when the pups were delivered was defined as postnatal day (PND) 0. Male and female rats were divided into control (C) and MS groups (n = 20‐24 in each group). The pups in the MS groups were moved to isolation cages at 0800 and placed on a heated pad maintained at 35°C for 240 minutes/d, from PND 2 to PND 11. This temperature was chosen to approximate the temperature of a dam. The rats were weaned on PND21 and housed at two rats per case. On PND 28, male rats were randomly selected from the C (n = 10) and MS (n = 11) groups and killed by decapitation, before their serum testosterone levels were measured.

### 2.2 Body weight

Throughout the experiments, the body weights of the rats were measured once a week from PND 1 to PND 91 in all groups.

### 2.3 Sexual maturation

The onset of vaginal opening (VO) in females and preputial separation (PS) in males, which are indicators of sexual maturation or the onset of puberty, was monitored daily from PND 29 to PND 40. Thereafter, the estrous cycle in females was monitored daily from PND 62 to PND 72 using vaginal smears.

### 2.4 Sexual behavior

Female rats were ovariectomized at 11 weeks of age, and sexual behavior was examined twice at 13 and 15 weeks of age. Estradiol (E2; 10 μg) was injected subcutaneously at 48 and 24 hours before the sexual behavior test, and progesterone (1 mg) was injected subcutaneously 4 hours before the test. The male rats did not receive any hormonal treatment prior to the sexual behavior test. Newly purchased male and female rats (9 weeks of age) were used as partners for the sexual behavior test. In the sexual behavior test, a pair of rats was put in a cage together after the male had been allowed 5 minutes' habituation in the cage, and sexual behavior was monitored via a video recording for 30 minutes.^7^ Mounting (the male places its forequarters on the hindquarters of the female from behind), intromission (mounting with vaginal insertion), and ejaculation (mounting with vaginal insertion and emission) were evaluated as male sexual behaviors,^16^ and proceptive behavior (hopping and darting, ear wiggling) and receptive behavior (the lordosis quotient [LQ]: lordotic responses/mounts × 100, and the lordosis rating [LR]: the mean number of lordosis reflexes in the first 10 mounts) were evaluated as female sexual behaviors.^17^ The mean values for each sexual behavior parameter were calculated at 13 and 15 weeks of age and used for the statistical analyses.

### 2.5 Blood sampling and hormone assays

At 15 weeks of age, the male rats were killed by decapitation, and then, their serum testosterone levels were measured. At 18 weeks of age, the female rats were killed by decapitation, and their serum luteinizing hormone (LH) and estradiol (E2) levels were measured. Serum was kept in a freezer at −20°C until the hormone assays. The serum LH concentration was measured using the I‐125 radioimmunoassay (RIA) kit (Rat LH [I‐125] RIA Kit; Institute of Isotopes Co., Ltd.).^12^ The analytical sensitivity of the LH assay was 0.1 mUI/mL, and the intra‐assay coefficient of variation was 6.5%.

The serum E2 level was measured using an electrochemiluminescence immunoassay (ECLIA, Elecsys E2 IV; Roche Diagnostics GmbH). The serum testosterone level was also measured using an ECLIA (Elecsys Testosterone II; Roche Diagnostics GmbH).

### 2.6 Statistical analysis

All data are presented as mean and standard error of the mean (SEM) values or as mean and standard deviation (SD) values. The Student *t* test, Mann‐Whitney *U* test, or chi‐square test was used for data comparisons. Statistical significance was defined as a *P*‐value of <.05.

## 3 RESULTS

**Figure 1:**
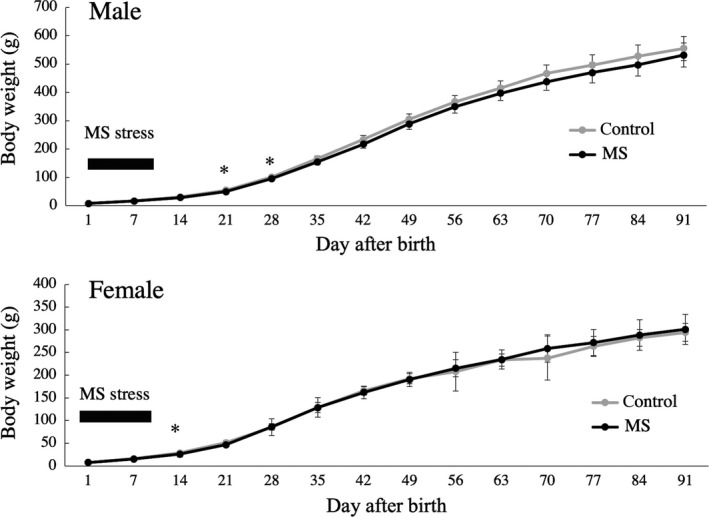
In both males and females, the body weights of the rats in the maternal separation (MS) group (n = 21‐23 per group) were significantly lower than those of the rats in the control (C) group on postnatal day (PND) 21, 28, and 14 (*P* < .05). Data are presented as mean ± SD values. *P < .05 vs the MS group or C group at the same time point

The body weights of the rats in the MS group were significantly lower than those of the rats in the C group on PND 21 and 28 in males and on PND 14 in females (Figure 1). Because body weight of MS group was not different from that in control group during stress loading period, temporary interruption of milk intake by MS might not directly affect nutritional condition and subsequent physiological changes.

**Figure 2:**
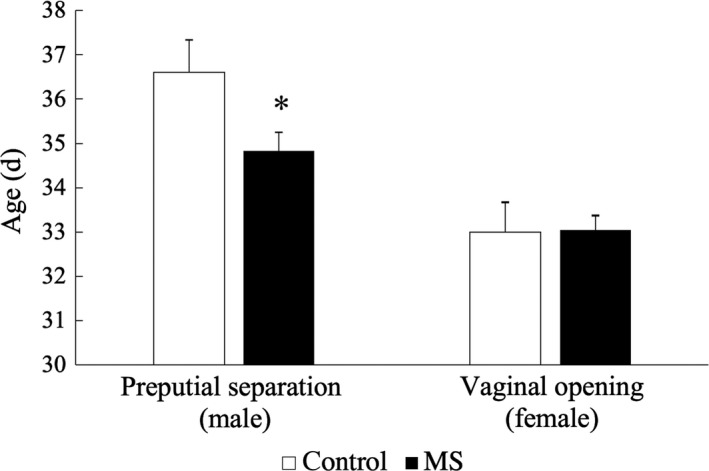
Preputial separation in male rats and vaginal opening in female rats are shown in the control (C) group and the maternal separation (MS) group (n = 10‐22 per group). Preputial separation occurred significantly earlier in the MS group than in the C group (*P* < .05). The timing of vaginal opening did not differ between the MS and C groups. Data are presented as mean ± SEM values. *P < .05 vs the MS group or C group

**Figure 3:**
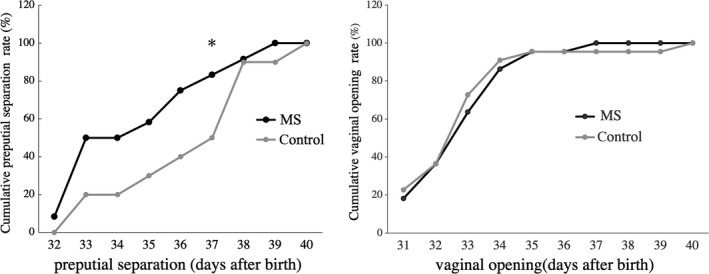
The number of male rats that exhibited preputial separation was greater in the maternal separation (MS) group than in the control (C) group on postnatal day (PND) 37 (χ^2^ test, *P* = .038; n = 10‐12 per group). The number of female rats that displayed vaginal opening did not differ between the MS and C groups from PND 31 to PND 40 (n = 22 per group)

**Figure 4:**
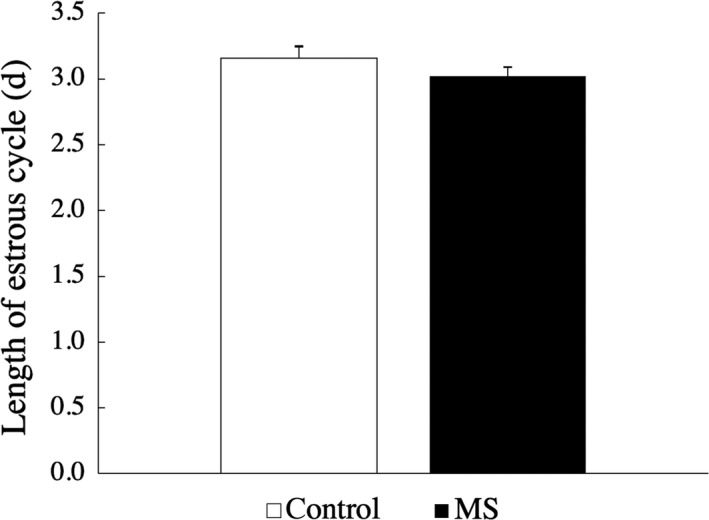
In female rats, the estrous cycle did not differ between the maternal separation (MS) and control (C) groups (n = 22 per group). Data are presented as mean ± SEM values

In the male rats, the onset of PS occurred significantly earlier in the MS group (34.83 ± 2.33 days of age) than in the C group (36.60 ± 2.32; Figure 2). The number of males that exhibited PS on PND 37 was greater in the MS group than in the C group (Figure 3). The serum testosterone level on PND 28 did not differ between the MS and C groups in the male rats. However, when the threshold (cutoff) level was defined as 1.30 ng/mL (the highest value in the C group), the number of rats that had elevated testosterone levels was significantly greater in the MS group than in the C group (Table 1). On the other hand, in the female rats the onset of VO did not differ between the MS (33.05 ± 1.53) and C (33.00 ± 1.95) groups (Figures 2 and 3). The estrous cycle also did not differ between these two groups (Figure 4).

**Table 1:** Male rats with or without elevated serum testosterone level on PND 28

Testosterone	<1.3 ng/mL not elevated	≧1.3 ng/mL elevated
Control	10	0
MS	7	4*

Note

Serum testosterone level on PND 28 was measured in the C group and the MS group (n = 10‐11 per group). We set a cutoff level in 1.3 ng/mL by upper distribution of control group. Number of animals that had elevated testosterone level in MS group was significantly larger than that in control groups (*P < .05, χ^2^ test,).

Abbreviations: MS, maternal separation; PND, postnatal day.

**Figure 5:**
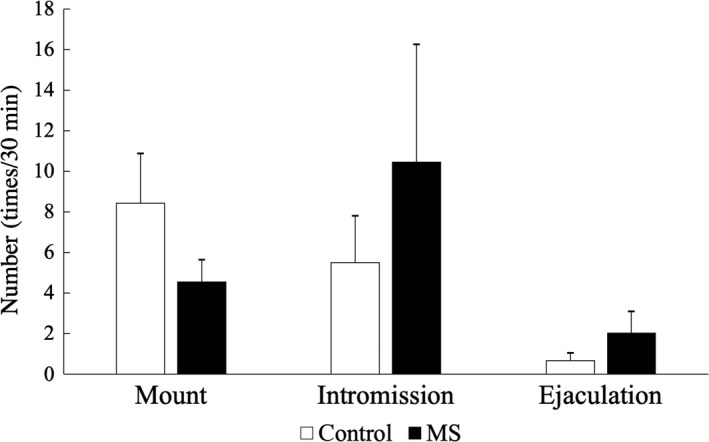
In male rats, sexual behavior did not differ between the two groups (n = 12 per group). The data represent the mean values obtained at 13 and 15 wk of age. Data are presented as mean ± SEM values

**Figure 6:**
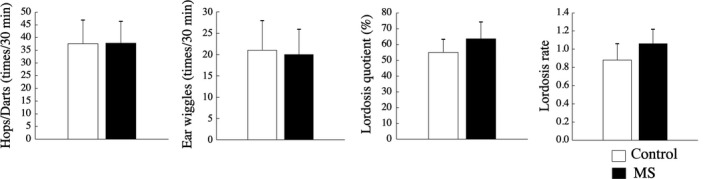
In female rats, sexual behavior did not differ between the two groups (n = 12 per group). The data represent the mean values obtained at 13 and 15 wk of age. Data are presented as mean ± SEM values

In males, the frequency of each type of sexual behavior did not differ between the MS and C groups: mounting (C: 8.42 ± 2.46 times/30 min; MS: 4.54 ± 1.10), intromission (C: 5.50 ± 2.32; MS: 10.46 ± 5.80), and ejaculation (C: 0.66 ± 0.39; MS: 2.04 ± 1.05; Figure 5). Similarly, in females the frequency of each type of sexual behavior did not differ between the MS and C groups: numbers of hops/darts (C: 37.58 ± 9.27 times/30 min; MS: 37.75 ± 8.60), number of ear wiggles (C: 21.04 ± 6.97; MS: 20.00 ± 5.92), LQ (C: 54.95 ± 8.21%; MS: 63.53 ± 9.84%), and LR (C: 0.88 ± 0.18; MS: 1.06 ± 0.16; Figure 6).

In males, the serum testosterone level at 15 weeks of age did not differ between the MS (5.92 ± 0.92 ng/mL) and C (6.53 ± 0.89) groups. In females, the serum LH level at 18 weeks of age did not differ between the MS (0.45 ± 0.03 ng/mL) and C (0.46 ± 0.03) groups. The serum E2 level at 18 weeks of age did not differ between the MS (35.70 ± 4.62 pg/mL) and C (31.20 ± 10.15) groups.

## 4 DISCUSSION

We and others have evaluated the long‐term effects of various kinds of stress in early life on sexual function in a rodent model, and different results were obtained according to the kind or timing of stress. In this study, we have shown that neonatal psychological stress induced by MS only advanced sexual maturation in male rats, resulting in elevated serum testosterone levels in the peripubertal period. On the other hand, the same stressor did not affect sexual maturation or estrous cyclicity in adulthood in female rats. Furthermore, we have also shown that neonatal psychological stress did not affect sexual behavior in either sex, at least under the present conditions. This is the first study to examine the long‐term effects of neonatal psychological stress on both sexual maturation and sexual behavior in adulthood in the same experiment.

In rodents, sexual maturation is affected by various kinds of stress in the prenatal and neonatal periods, but the reported effects of such stress differ according to the kind or timing of stress. For example, prenatal metabolic stress (maternal undernutrition) and neonatal immune stress (the injection of lipopolysaccharides [LPS]) delayed PS in male rats and VO in female rats,^6^, ^7^, ^8^, ^11^, ^12^, ^18^ indicating that these stressors disturb sexual maturation in both sexes. Similarly, subjecting mothers to psychological stress during pregnancy delayed PS in male offspring in rats, indicating that subjecting mothers to prenatal stress disturbs sexual maturation in male offspring.^19^ However, as for neonatal psychological stress, MS delayed PS in male rats in a previous study,^15^ but advanced it in the present study. This is the first report that male rat's sexual maturation was accelerated. It is difficult to explain this discrepancy based on differences in the experimental conditions because the duration of stress (PND 1 to PND 14 in the previous study and PND 2 to PND 11 in the present study) and the severity of the stress (3 and 4 hours/d, respectively) did not differ markedly between the studies. However, our data were supported by serum testosterone level data. Testosterone plays an essential role in sexual maturation in male rodents.^20^ In our study, the number of male rats with elevated serum testosterone levels in the prepubertal period was greater in the MS group. Therefore, the advanced sexual maturation seen in the male rats in the MS group seemed to have been induced by advanced testosterone secretion in the prepubertal period. In addition, it has been shown that kisspeptin and gonadotropin‐releasing hormone (GnRH) signaling are required for neonatal testosterone secretion in male mice.^21^ It is speculated that the upregulation of kisspeptin expression occurred earlier in the males in the MS group, resulting in the earlier onset of testosterone secretion and sexual maturation. If this were true, the epigenetic modulation of hypothalamic neurons might be involved in the long‐term effects of neonatal psychological stress on male sexual maturation. On the other hand, neonatal psychological stress did not affect VO/estrous cyclicity in females in either a previous study^15^ or the present study. In addition, it is suggested that psychological stress and stress in the neonatal period have weaker long‐term effects on sexual maturation than other stressors and stress in the prenatal period.

The long‐term effects of prenatal and neonatal stress on sexual behavior differ among stressors and/or between sexes, as is the case for sexual maturation. The immune stress induced by the injection of LPS seems to have the strongest effect on sexual function because it has long‐term suppressive effects on both sexual maturation and sexual behavior in both sexes. Namely, prenatal immune stress disrupted both sexual maturation and sexual behavior in both sexes, and it also enhanced stress responses in mice.^22^, ^23^, ^24^ Neonatal immune stress also had the same long‐term effects as prenatal immune stress on sexual functions in male and female rats.^11^, ^12^ As for metabolic stress, we and others have reported that prenatal undernutrition disrupted sexual maturation in both sexes, whereas it only disrupted sexual behavior in females.^7^, ^8^ As for neonatal psychological stress, it was reported that male sexual behavior was not affected by MS,^15^ and in the present study, we have added female data and reported that neonatal psychological stress did not affect sexual behavior in either sex. We previously found that the disrupted sexual behavior seen in adulthood after certain forms of early‐life stress, such as prenatal undernutrition in females and neonatal immune stress, was associated with reduced hypothalamic expression of the progesterone receptor (PR) in both sexes.^8^, ^12^ Furthermore, male rats that were subjected to prenatal undernutrition exhibited normal sexual behavior and normal hypothalamic PR expression,^7^ and in the present study, the male and female rats also displayed normal sexual behavior and normal hypothalamic PR expression (data not shown). Hence, a consistent association between sexual behavior and PR expression has been reported; that is, it was either reduced or normal, which indicates that the disruption of sexual behavior occurs due to reduced hypothalamic PR expression. In addition, it seems that psychological stress and/or stress in the neonatal period have weaker long‐term effects on sexual behavior and sexual maturation than other kinds of stress and/or stress in the prenatal period. It should be emphasized that stress in the prenatal and neonatal periods has long‐term effects on sexual function and that these effects differ according to the kind of stress, the period of exposure, and/or sex. As sexual behavior is controlled by the central nervous system (CNS) and early‐life stress could result in the epigenetic modulation of brain neurons, the abovementioned discrepancies regarding the long‐term effects of stress on sexual behavior might be explained by variations in the epigenetic modulation induced in response to stress in early life. Neonatal psychological stress might not have a strong effect on the epigenetic modulation of hypothalamic neurons, which are involved in sexual behavior.

Histone acetylation and DNA methylation play key roles in the regulation of gene expression through epigenetic mechanisms.^25^ An appropriate epigenetic landscape is essential for neuronal function, and environmental factors are known to affect histone acetylation and DNA methylation of the brain during early development. Therefore, the long‐term effects of stress in early life on physical functions in later life might be mediated by epigenetic mechanisms in the CNS. In fact, in mice MS in the neonatal period caused persistent hypomethylation of the promoter region of the vasopressin gene in the paraventricular nucleus.^26^ It was also reported that MS in the neonatal period caused hypomethylation of the corticotropin‐releasing hormone (CRH) promotor area and hypothalamic pituitary adrenal axis hypersensitivity.^27^ This epigenetic modulation could result in the long‐lasting upregulation of CRH transcriptional activity in the hypothalamic paraventricular nucleus. Prenatal metabolic stress also decreased DNA methylation of the CRH promotor area in rats, which resulted in enhanced CRH expression.^28^ In the present study, MS disrupted sexual maturation in male rats. Furthermore, MS temporarily reduced the body weights of both male and female rats. In particular, temporary weight reduction was observed in males after MS, but sexual maturation was only influenced (accelerated) by MS in males. These effects might be explained by long‐lasting epigenetic modulation of hypothalamic neurons due to neonatal psychological stress. The pathophysiological mechanisms responsible for the responses of weight reduction and sexual maturation to early‐life psychological stress in each sex remain to be clarified.

In conclusion, the neonatal psychological stress induced by MS only advanced sexual maturation in male rats, and increased testosterone secretion might be involved in such changes. These results indicate that the long‐term effects of neonatal psychological stress on sexual maturation might exhibit sexual dimorphism. However, we could not confirm the underlying mechanisms and pathophysiological roles of this sexual dimorphism, and some further examinations would be needed in future studies. On the other hand, neonatal psychological stress did not affect sexual behavior in adulthood in either sex. Preventing psychological stress, such as violence, abuse, and neglect, in the neonatal period is important for the normal development of physical functions, including sexual functions.

## DISCLOSURES

*Conflict of interest*: The authors declare that no conflicts of interest exist. *Human and animal rights*: This article does not describe any experiments involving human participants. All of the institutional and national guidelines for the care and use of laboratory animals were followed. The protocol for the research project was approved by a suitably constituted ethics committee.
